# How to manage various arrhythmias and sudden cardiac death in the cardiovascular intensive care

**DOI:** 10.1186/s40560-018-0292-x

**Published:** 2018-04-11

**Authors:** Yoshinori Kobayashi

**Affiliations:** Division of Cardiology, Tokai University Hachioji Hospital, 1838 Ishikawa-machi Hachioji-shi, Tokyo, 192-0032 Japan

**Keywords:** Cardiovascular intensive care, Arrhythmias, Electrical storm, Acute myocardial infarction, Congestive heart failure

## Abstract

In the clinical practice of cardiovascular critical care, we often observe a variety of arrhythmias in the patients either with (secondary) or without (idiopathic) underlying heart diseases. In this manuscript, the clinical background and management of various arrhythmias treated in the CCU/ICU will be reviewed.

The mechanism and background of lethal ventricular tachyarrhythmias vary as time elapses after the onset of MI that should be carefully considered to select a most suitable therapy. In the category of non-ischemic cardiomyopathy, several diseases are known to be complicated by the various ventricular tachyarrhythmias with some specific mechanisms.

According to the large-scale registry data, the most common arrhythmia is atrioventricular block. It is essential for the decision of permanent pacemaker indication to rule out the presence of transient causes such as ischemia and electrolyte abnormalities.

The prevalence of atrial fibrillation (AF) is very high in the patients with heart failure (HF) and myocardial infarction (MI). AF and HF have a reciprocal causal relationship; thus, both are associated with the poor prognosis. Paroxysmal AF occurs in 5 to 20% during the acute phase of MI and triggered by several specific factors including pump failure, atrial ischemia, and autonomic instability.

After the total management of patients with various arrhythmias and basic heart diseases, the risk of sudden cardiac death should be stratified for each patient to assess the individual need for preventive therapies.

Finally, it is recommended that the modalities of the treatment and prophylaxis should be selected on a case-by-case basis in the scene of critical care.

## Background

**Fig. 1:**
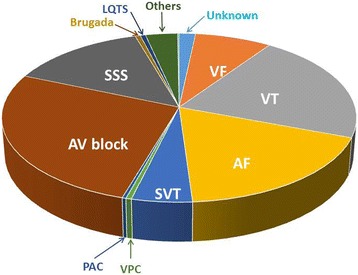
The contents of the arrhythmias in the patients who were admitted to the CCU/ICU in the Tokyo CCU Network for the treatment of arrhythmias in 2014. Those correspond to approximately 10% of the total patients. The most common arrhythmia was AV block, which was followed by ventricular tachycardia and atrial fibrillation

According to the registry of the Tokyo CCU Network of the patients hospitalized in the cardiovascular intensive care units (CCU/ICU) of 72 leading hospitals capable of advanced cardiovascular care in the Tokyo metropolitan area, approximately 10% received intensive care due to a variety of arrhythmias as a main cause of their admission. The arrhythmias were mainly comprised of idiopathic bradyarrhythmias, including atrioventricular conduction disturbances and sinus node dysfunction (Fig. 1 and Table 1), followed by ventricular tachycardia (VT) and atrial fibrillation (AF).

**Table 1:** Contents of arrhythmias as causes of admission

Cardiac arrest	22	
Ventricular fibrillation (VF)	103	
Ventricular tachycardia (VT)	277	
Sustained VT		198
Non-sustained VT		68
Torsade de points (TdP)		11
Atrial fibrillation (AF)	238	
Supraventricular tachycardia (SVT)	64	
Ventricular premature contraction(VPC)	8	
Atrial premature contraction (APC)	4	
AV block	351	
1st degree		1
2nd degree		65
Wenckebach		17
Morbitz II		48
3rd degree		285
Sick sinus syndrome (SSS)	184	
Brugada syndrome	7	
Long QT syndrome	7	
Others	43	
Unknown	2	

Further, we have a lot of patients transferred to the critical care for severely ill conditions such as cardiogenic shock or severe heart failure (HF) due to an acute myocardial infarction (MI) and/or an advanced stage of various cardiomyopathies. Such patients frequently have various arrhythmias that should be controlled to improve their cardiac performance and to decrease the prevalence of sudden cardiac death (SCD).

In this chapter, the clinical background and management of various arrhythmias treated in the cardiovascular critical care unit will be reviewed. The risk stratification and therapeutic strategies for the prevention of SCD will also be described.

## Clinical features and management of VT/VF and electrical storms (ESs) in the CCU/ICU

About one fourth of the patients who are admitted to the CCU/ICU for the management of arrhythmias receive a diagnosis of VT or ventricular fibrillation (VF). They have a variety of underlying heart diseases, including ischemic heart disease (IHD) and various cardiomyopathies. Using the database of the Tokyo CCU Network between 2012 and 2014, there were 1067 patients who were admitted to the CCU for the management of VT/VF as a main clinical manifestation [1]. Among them, 312 patients (29.2%) had IHD, 88 (8.2%) dilated cardiomyopathy (DCM), 78 (7.3%) hypertrophiccardiomyopathy (HCM), 25 (2.3%) cardiac sarcoidosis, and 18 (1.7%) arrhythmogenic right ventricular cardiomyopathy. However, approximately 40% of the patients diagnosed with idiopathic VT and idiopathic VF had no structural abnormalities found during the clinical check-ups. In this chapter, VT/VF and electrical storms (ESs) associated with and without structural heart disease, particularly during the acute phase of an MI, will be focused on.

### VT/VF and ESs associated with acute MIs

Life-threatening ventricular tachyarrhythmias (VTAs), including VT and VF, can occur anytime from during the super-acute phase to during the remote phase of an MI. From the old days, the animal experimental studies such as the canine MI model (Harris Model) have shown that the characteristics and mechanisms of the VTAs dramatically vary as time elapses after the onset of an MI [2, 3]. Such a temporal variation in the mechanism of the VTAs obtained by experimental studies cannot totally be extrapolated to VTAs during an acute or subacute MI in humans, because there is a greater number of factors affecting the occurrence of VTAs in clinical practice as compared to the coronary ligation model. Those are spontaneous and intentional reperfusion and iatrogenic factors. Using the large data from the Tokyo CCU Network, we have elucidated the incidence, clinical features, background, and prognosis of patients with life-threatening VTAs during the acute or subacute phase of an MI and the time interval-dependent difference from the onset of the MI [4]. We analyzed the registry data from years 2011 and 2012 undertaken specifically in MI patients. The detailed individual data were provided from the allied hospitals for 2811 patients for 2011 and 3192 patients for 2012. After the perusal of the individual data, we judged that a total of 160 patients (141 males and 19 females, average age 66 ± 12 years) experienced ESs, either before or after the hospitalization, during the acute or subacute phase of an MI, if an ES was defined as two or more recurrent sustained VTAs during a 24-h period. The incidence of an ES was 160/6003 patients (2.67%). Among those, in 133 patients, the precise time of the onset of the MI could be obtained. Those 133 patients were then divided into three groups according to the time interval from the onset of the MI to the first episode of the VTA, that is, (1) the super-acute phase of the MI (MI-VTA interval ≤ 1 h: group A 63 patients), (2) acute phase of the MI (1 h < MI-VTA interval ≤ 24 h: group -B 51 patients), and (3) subacute phase of the MI (MI-VTA interval > 24 h: group C 19 patients). We also compared the demographic data and clinical parameters among those three groups (Table 2). In group A, the majority of the patients had ESs outside of the hospital before admission, whereas the ESs occurred in the catheter laboratory in the majority of patients in group B. On the other hand, the ESs emerged either in the CCU or general ward in group C. In the group A and group B patients, the main arrhythmia observed was VF, while it was VT in group C. In group A, the ESs were obviously associated with a large infarction size and severe hemodynamic deterioration, leading to a poor in-hospital mortality. In group B, the background of the ES patients varied rather widely, with a similar number of patients with a Killip-I and Killip-IV class and also anterior and inferior infarctions. The in-hospital mortality was relatively low as compared to that of group-A and group-C, however, it was still worse than that in the patients without ESs (6%). Finally, in group C, the status of the patients was not so severe on admission, in that there was a greater percentage of Killip-I patients and a relatively low peak-CK. Most of the ESs occurred during the subacute phase of the MI. The ESs themselves were more severe as the number of DC shocks applied were significantly greater than that in group A and group B. The short-term prognosis was worst in group C. As such, the background of the ESs was multi-factorial, but there were some periodic differences in the patient characteristics and contents of the arrhythmias.

**Table 2:** Comparison of clinical background among 3 groups

	Group A	Group B	Group C	Statistics
	MI-ES interval 0–1 h	Interval 1–24 h	Interval > 24 h	
Patient no.	63	51	19	
Average age (years)	65 ± 11	65 ± 13	71 ± 12	*P* < 0.1
Average MI-ES interval (hours)	0.3 ± 0.4	4.8 ± 5.1	204 ± 160	*P* < 0.001
Place of ES				
Out of hospital	54	10	0	*P* < 0.001
Emergency room	4	8	0	
Catheter laboratory	3	27	0	
CCU	2	6	16	
General ward	0	0	3	
Ml site(9^6)	Anterior 75%	Anterior 52%	Anterior 74%	*P* < 0.01
	Inferopost 25%	Inferopost 48%	Inferopost 26%	
Killip class (pts no.)	I, 12; II, 6	I, 15; II, 6	I, 9; II, 4	*P* < 0.01
	III, 4; IV, 32	III, 7; IV, 17	III, 3; IV, 3	
Peak CK	6810 ± 6110	5780 ± 3700	3170 ± 3180	
Arrhythmia contents ES				
Cardiac arrest	3	1	0	
PEA	9	0	0	
VF only	13	22	3	
VF and VT	32	21	7	
VT only	4	7	9	
Number of DC	4.2 ± 2.5	4.3 ± 3.2	9.5 ± 14.0	*P* < 0.01
In-hospital mortality	49.2%	33.3%	57.9%	*P* < 0.01

*MI* myocardial infarction, *ES* electrical storm, *Pts* patients, *PEA* pulseless electrical activity, *DC* direct current shocks, *inferopost* inferoposterior

**Fig. 2:**
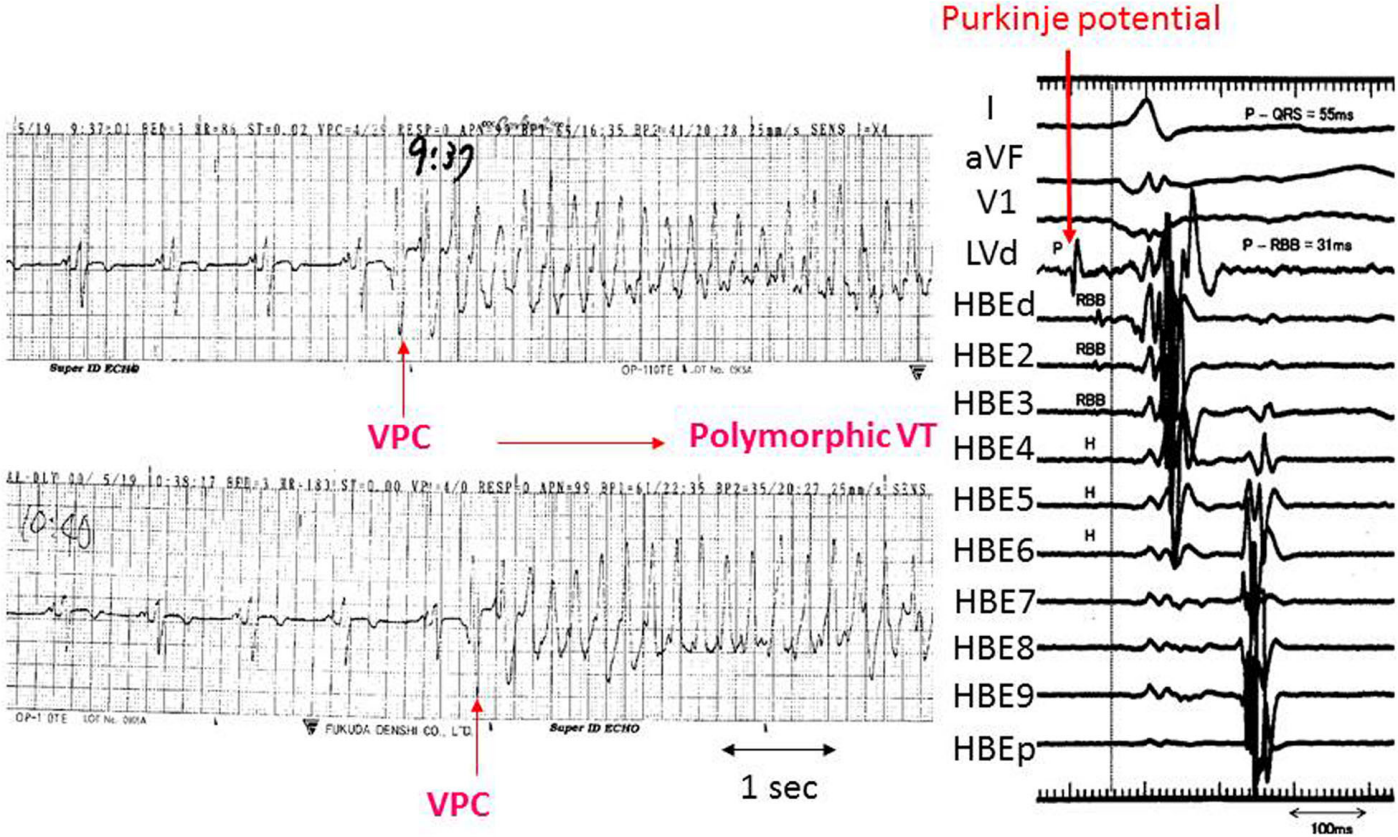
A case (67 years old, male) with a VT/VF storm that emerged during the acute phase of an anterior infarction (4th day). Left panel: The monitored ECG recording revealed that this polymorphic tachycardia was always initiated by PVCs with exactly the same QRS morphology with a relatively narrow configuration. Right panel: Detailed LV mapping demonstrated that the Purkinje potentials (indicated by the red arrows) from the posterior fascicular region preceded the onset of the QRS complex by 55 ms during the PVCs. HBE His bundle electrogram, P Purkinje potential, RBB right bundle branch potential, H His potential

Beta blockers [5], amiodarone [6], and nifekalant (a pure Ikr blocker) [7] have been shown to be effective in suppressing ESs during an acute MI. We often experience drug-refractory recurrent VTAs in patients with hemodynamic deterioration. For such patients, intra-aortic balloon pumping is a potent non-pharmacological therapy applied as the first choice and has been shown to be effective in suppressing ESs, probably by virtue of the improvement in both the hemodynamics and coronary perfusion [8]. For the patients complicated with cardiogenic shock, hypoxia due to severe pulmonary edema, and cardiac arrest, percutaneous cardiopulmonary support (PCPS) is also introduced [9]. There have been several reports in which a satellite ganglion block and renal sympathetic nerve ablation may have been effective in suppressing the ES [6, 10]. When an ES could not be suppressed by drug therapy and cardiac support devices, catheter ablation procedures have occasionally been applied to rescue patients [11, 12]. In that case, a ventricular premature complex (VPC) triggering polymorphic VT or VF is one of the targets of the ablation. The triggering VPCs commonly originate from the surviving Purkinje network exhibiting a relatively narrow QRS configuration (Fig. 2). Radiofrequency deliveries at the earliest activation site where the local Purkinje potential precedes the QRS complex during the VPC usually result in the successful elimination of the incessant VTA.

### VT/VF and ESs associated with non-ischemic heart disease

There are a variety of heart diseases in this category of patients, which is known to be complicated by VT and VF (Table 3). Accordingly, there are multiple electrophysiologic mechanisms of VT, including scar-related reentry (channel-dependent and isthmus-dependent), His bundle-Purkinje-related reentry (bundle-branch reentry, inter-fascicular reentry, and intra-fascicular reentry), and focal tachycardia (enhanced automaticity and triggered activity). It has been considered that bundle-branch reentrant tachycardia (BBRT) is a specific arrhythmia observed in patients with DCM, whereas it is rarely observed in those with IHD. However, recent reports have clearly showed that this mechanism similarly causes the VTs in both categories of basic heart disease [13, 14]. During the advanced stage of non-ischemic CM, we sometimes experience multiple morphologies of the QRS complex that transform spontaneously or during pacing maneuvers. Such VTs, so-called “pleomorphic VTs,” are attributable to complex degenerative ventricular lesions leading to the formation of multiple channels of slow conduction [15, 16]. The culprit lesions for sustained VTs have also been shown to more likely be located at epicardial sites in DCM as compared to IHD [16]. In patients with HCM, polymorphic VT or VF is a more common arrhythmia than monomorphic VT. Monomorphic VT is commonly observed in patients with an apical ventricular aneurysm formation resulting from a long-term mid-ventricular obstruction [17].

**Table 3:** Basic heart disease categorized in the non-ischemic heart disease and known to be complicated by VTAs

1) Degenerative disease	
a) Dilated cardiomyopathy (DCM)	
b) Arrhythmogenic right ventricularcardiomyopathy (ARVC)	
2) Inflammatory disease	
a) Acute myocarditis	
b) Chronic myocarditis	
c) Cardiac sarcoidosis	
3) Hypertrophic disease	
a) Hypertrophiccardiomyopathy (HCM)	
b) Cardiac amyloidosis	
4) Congestive heart disease and post-surgery (Tetralogy of Fallot)	
5) Mitral valve prolapse	
6) Pseudo ventricular aneurism	
7) Neuro-muscular disease (myotonic dystrophy)	

Cardiac sarcoidosis (CS) is observed with a greater prevalence in Japanese people (20%) than Caucasians and black Americans (2%) [18]. The prevalence of subclinical CS diagnosed by an autopsy study was also 70–80% in Japanese and 20% in Caucasians and black Americans, respectively. CS is complicated by various arrhythmias. The most common arrhythmia is AV block, followed by VT. A recent report showed that VT storms sometimes emerge in CS, particularly after the introduction of steroid therapy [19]. Most of the VTs associated with CS are due to scar-related reentry, which is located in the interventricular septum, right ventricular, or entire LV with patchy scarring. Storms are shown to be successfully suppressed by catheter ablation; however, the recurrence rate is relatively high (30–40% per year) [20].

In VTAs associated with non-ischemic CM, the first-line pharmacological therapy is amiodarone; however, the largest trial to date, that is, the Sudden Cardiac Death in Heart Failure Trial (SCD-HeFT), showed no significant difference in the mortality between the amiodarone treatment group and placebo group [21]. On the basis of that trial, it has been recommended that amiodarone not be used routinely in patients with DCM unless a specific arrhythmia indication exists [22]. Amiodarone is known to lengthen the tachycardia cycle length and reduce the frequency of implantable cardioverter defibrillator (ICD) shocks without worsening HF. Beta blockers are also shown to improve the prognosis of patients with DCM reducing both heart-failure-related death and sudden cardiac death [23, 24], and therefore, beta blockers are considered one of the standard medications for DCM. However, the introduction of those drugs should be done carefully in patients with severe HF because of their negative inotropic effects.

In patients with non-ischemic CM, aggravation of HF is usually the predisposing factor of the occurrence of arrhythmias and VT/VF storms. Therefore, the therapeutic target should simultaneously be addressed to improve the HF. That includes pharmacological therapies (diuretics, vasodilators, inhibitors of the renin-angiotensin system, positive inotropic agents, etc.) and non-pharmacological modalities (left ventricular assist devices, biventricular pacing, etc.) [25].

### Idiopathic VT

As mentioned before, approximately 40% of patients who are admitted to the CCU/ICU for the management of VT are found not to have structural heart disease by screening check-ups [1]. Except for verapamil sensitive left VT (so-called fascicular VT), which has been shown to be caused by a reentrant mechanism probably involving the Purkinje network, idiopathic VT (IVT) commonly occurs due to a focal mechanism. The origins of focal IVTs are distributed in a variety of areas of the right (RV) and left (LV) ventricles. The most common site of origin is the outflow tract region of both the RV and LV. The mitral and tricuspid annular regions and papillary muscles are also the next most common sites of IVT origins [26].

Most IVTs usually present with a hemodynamically stable condition upon admission; however, IVT can occasionally appear as an unstable rapid VT in which prompt DC cardioversion is needed. Otherwise, drug therapy is the first choice to bail out any incessant form of VTs and for prophylactic purposes.

Verapamil is the most effective drug for the fascicular VT, which has a relatively narrow QRS configuration with CRBBB and both a superior axis (originating from the posterior fascicle) and inferior axis (originating from the anterior fascicle). There is another type of fascicular VT, that is, the upper septal type, which is reported to have a very narrow QRS complex with a QRS width of less than 120 ms. Catheter ablation can cure these tachycardias with a high success rate (> 90%) [27].

Furthermore, beta blockers are the first choice of drugs for focal IVTs following the administration of non-dihydropyridine calcium channel blockers such as verapamil. Class I and III drugs are also shown to be effective for focal IVTs [28]. Even though catheter ablation is also a very effective tool to eliminate these tachycardias, the consequence of the procedure deeply depends on the site of origin. The success rate for RV outflow origins is relatively high, while that for LV summit, papillary muscle, and so-called LV Crux VTs has not reached a satisfactory level [26, 28]. Therefore, the precise identification of the origin during the pre-ablation stage, while carefully examining the QRS morphology, is essential for a successful ablation. There are several diagnostic algorithms to determine the site of origin using the 12 lead ECG [29, 30].

### QT prolongation and torsades de pointes (Tdp) polymorphic VT

The patients who are admitted to the ICU/CCU usually have several risk factors that can predispose them to QT prolongation and Tdp tachycardias [31]. Those include an elderly age, underlying heart disease (particularly MIs), the presence of HF, renal and hepatic dysfunction, electrolyte abnormalities, bradycardias, and various drugs such as diuretics, antiarrhythmic agents, and sedative agents that facilitate QT prolongation and hypokalemia (Table 4). It has been shown that a greater risk for the development of Tdp in the hospital setting occurs with the clustering of multiple recognizable risk factors in a single patient [31, 32].

**Table 4:** Risk factors and drugs causing torsade de pointes in hospitalized patients

Clinically recognizable risk factors	List of drugs causing torsade de points
1) QTc > 500 ms	1) Antiarrhythmicdrugs
2) Use of QT-prolonging drug	i) Class Ia agents (disopyramide, cibenzoline)
3) Structural heart disease AMI and CHF	ii) Class III agents (amiodarone, bepridil, nifekalant)
4) Advanced age	2) Antidepressant (amitiptyline, desipramide)
5) Female sex	3) Antipsychotic agents (chlorpromazine, haloperidol)
6) Hypokalemia	4) Anticonvulsant (felbamate, fosphenytoin)
7) Hypomagnesemia	5) Sedative agents (droperidol)
8) Hypocalcemia	6) Antihistamine agent (astemizole, terfenadine)
9) Treatment with diuretics	7) Antibiotics (clarithromycin, erythromycin)
10) Impaired hepatic drug metabolism	8) Antiviral agents (foscarnet)
11) Bradycardia	9) Antimalarial agents (halofantrine, pentamidine)
Clinically silent risk factors	10) Antihypertensive agents (isradipine, nicardipine)
1) Latent congenital LQTS	11) Anticancer agent (tamoxifen, arsenic trioxide)
2) Genetic polymorphism	12) Anti-migraine agents (naratriptan, zolmitriptan)
	13) Lipid-lowering agent (probucol)

**Fig. 3:**
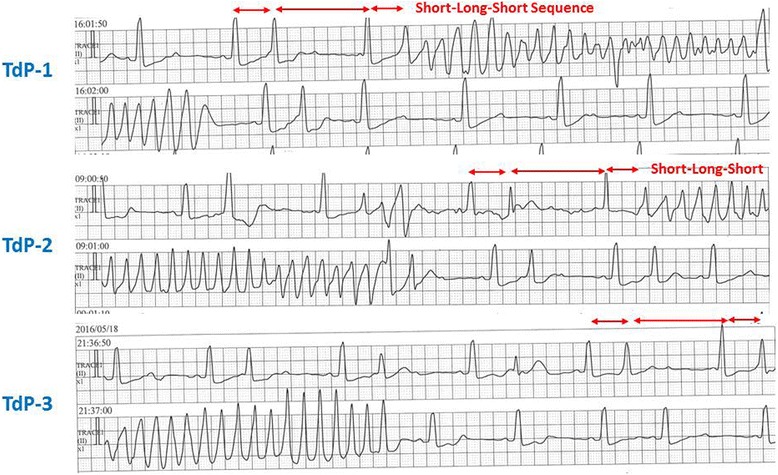
Monitored ECG recordings (three episodes) showing a torsades de pointes (Tdp) tachycardia in a patient with an AV conduction disturbance and hypopotassemia (83 year old, female). Each episode of the Tdp tachycardia was preceded by a short-long-short sequence of the R-R intervals created by isolated ventricular premature contractions

The ECG signs as predictors of Tdp are (1) a QTc interval > 500 ms, (2) macroscopic T wave alternans, and (3) a prolonged QT interval with an increase in the terminal portion of the T wave (*T*^peak^ − *T*^end^ interval) [31, 33]. Prior to the development of Tdp, a typical short-long-short sequence of the R-R interval is often observed with a marked QT prolongation and T-U wave distortion with the last sinus beat (after the long pause) (Fig. 3).

## Clinical features and management of bradyarrhythmias in the CCU/ICU

### Atrioventricular (AV) block

AV block is the most common arrhythmia in critical care medicine. As shown in Fig. 1, approximately 27% of patients with various arrhythmias as the main cause of their admission to Tokyo CCU Network hospitals were due to AV block. Among them, most of the patients (81%) were diagnosed with complete AV block. The majority of the remaining patients had Mobitz type II second-degree AV block, while Wenckebach type second-degree AV block was relatively rare. The background of the appearance of AV block should be evaluated individually, because multiple factors may be associated with the AV conduction disturbance, including acute ischemia, chronic ischemic heart disease (IHD), degenerative disease, acute inflammatory disease (particularly fulminant myocarditis), chronic inflammatory disease (such as cardiac sarcoidosis), electrolyte disturbance (such as hyperkalemia), and the use of drugs suppressing the AV conduction, including Ca channel blockers, beta blockers, digitalis, and class I and III antiarrhythmic agents. In some patients, AV block seems to occur due to multiple factors. On the other hand, there are more patients in whom no cause of the AV block could be found, which is so-called idiopathic AV block (progressive cardiac conduction disease), which has been linked to a strong genetic background, i.e., gene mutations involving SCN5A and SCN1B [34]. It is essential in these patients, with obvious transient and reversible causes behind the AV block, to identify and improve, or eliminate, those causes by correcting any electrolyte abnormalities, cessation of the offending drugs, treatment of myocardial ischemia, and so on.

**Fig. 4:**
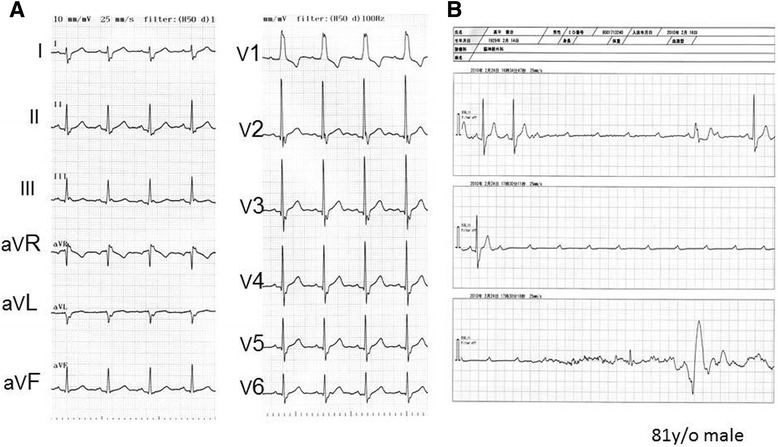
A representative case of paroxysmal AV block (81 years old, male). **a** 12-lead ECG before the syncope. **b** The monitor ECG during a syncopal episode in the CCU (for further explanation, see the text)

In the CCU/ICU, we occasionally experience paroxysmal AV block that is characterized by an abrupt and sustained AV block, usually in the absence of structural heart disease [35]. It is also commonly associated with long episodes of ventricular asystole resulting in syncope and even SCD. As an example, in the representative male case, shown in Fig. 2, he previously experienced several episodes of syncope, and the most recent episode caused a traumatic subarachnoid hemorrhage rendering the patient being admitted into the ICU. Before that episode, the ECG exhibited complete right bundle branch block; however, the PR interval was normal and a slight right anterior deviation was observed (Fig. 4a). The monitored ECG during the syncopal episode revealed the sudden onset of complete AV block without any escape rhythm (a long pause) (Fig. 4b). Paroxysmal AV block has been shown to be a unique phenotype of an infra-Hisian conduction disturbance. Because this is a rare and abrupt phenomenon, the diagnosis is sometimes difficult even when utilizing long-term Holter recordings and loop recorders. An electrophysiologic (EP) study with a provocation attempt using class I antiarrhythmic agents may play some role in the diagnosis of this entity [35].

### Sinus node dysfunction (SND)

Out of the patients who are admitted for treating arrhythmias, approximately 14% are due to sick sinus syndrome (Fig. 1). As compared with AV block, SND is more of an intrinsic disorder localized to the sinus node and surrounding atrial tissue resulting in a variety of bradyarrhythmias (sinus bradycardia, sinus pauses, sinoatrial block, and tachycardia-bradycardia syndrome). The extrinsic factors facilitating SND include drug effects, an excessive vagal tone, electrical abnormalities, sleep apnea, and hypothyroidism [36]. Because most occurrences of SND become gradually more aggravated with the progression of idiopathic degenerative disorders, the patients are often asymptomatic or have only mild symptoms. The SND patients who are admitted into the CCU/ICU commonly have severe symptoms such as syncopal attacks and collapsing. Syncope is most likely to occur in patients with tachycardia bradycardia syndrome with prolonged sinus pauses.

### Temporary pacing and permanent pacemaker implantations

Temporary pacing is sometimes required in patients with an acute MI. According to the guidelines for the management of an ST-Elevation Myocardial Infarction (STEMI), pacing is indicated (class I) for complete AV block, symptomatic bradyarrhythmias refractory to drug therapy, and tri-fascicular block, including alternating bundle branch block and bifascicular block with Mobitz type II second-degree AV block [37].

For patients with symptomatic AV block in the absence of a transient cause of the AV conduction disturbance, a permanent pacemaker (PM) is usually implanted. In patients with an acute MI, the necessity of a permanent PM is not that high, as the incidence of a PM implantation is shown to be needed in less than 1% of the total acute MI patients. Most AV block (even a high-degree block) has a transient nature, appearing only for a short term during the acute phase of an MI and is associated with an inferior MI and vagotony. Candidates for a PM implantation usually have an infra-Hisian block associated with an anterior MI rather than an inferior MI [38].

## Clinical features and management of AF in the CCU/ICU

AF is also one of the common arrhythmias observed in cardiovascular critical care. Approximately 18% of patients with various arrhythmias who are admitted to the CCU/ICU have AF (Fig. 1). AF is also frequently seen in the setting of HF and an MI (10–49%). In addition, a variety of pathogenic factors are associated with AF in clinical practice as shown in Table 5.

**Table 5:** Pathogenic factors associated with AF occurrence in critical care medicine

1) Heart failure: HFrEF_;_ HEpEF
2) Cardiac ischemia: myocardial infarction
3) Inflammation: pericarditis myocarditis sepsis
4) Cardiac intervention: after cardiac surgery
5) Respiratory disorder: COPD
6) Bradyarrhythmias: sinus node dysfunction, post-PM implantation
7) Neuro-humoral imbalance: hyperthyroidism, heart failure, dehydration
8) Drug-induced: cathecolamine, teophylline, cilostazol, etc.
9) Intoxication: alcohol, CO
10) Chronic kidney disease (CKD)

### AF associated with HF

**Fig. 5:**
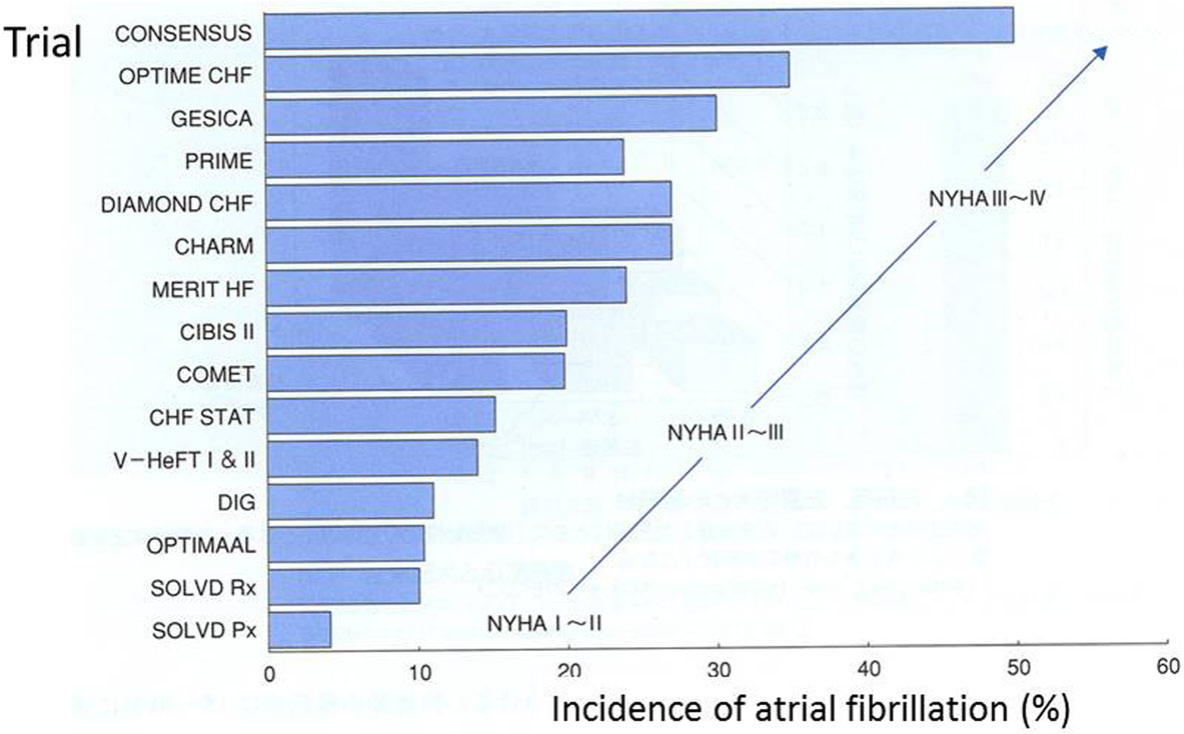
The relationship between the severity of the CHF and the prevalence of AF. The data were collected from randomized trials of patients with CHF with various severities of heart failure (NYHA classification). The prevalence of AF is well correlated with the severity of CHF (cited from reference [39])

In patients with HF, various factors, including a volume overload in the atrium, increased intra-atrial pressure, hypoxia, and a neuro-humoral imbalance, contribute to the occurrence of arrhythmias. The severity of the HF has been shown to be well correlated with the prevalence of AF [39], and that prevalence in New York Heart Association (NYHA) class IV patients is more than 50% (Fig. 5), while that in class II patients is only 10–15%. Recently, the physicians’ attention has been directed to HF associated with a preserved ejection fraction (HFpEF), particularly its pathophysiology, background, and prognosis. Campbell et al. [40] demonstrated in their review article on the previous clinical trials, evaluating the effects of various drug interventions on the outcome of HF patients, that the prevalence of AF is similar between the HFpEF patients and patients with HF with a reduced ejection fraction (HFrEF). Thus, similar to systolic dysfunction, diastolic dysfunction is also shown to be an important factor underlying the occurrence of AF.

**Fig. 6:**
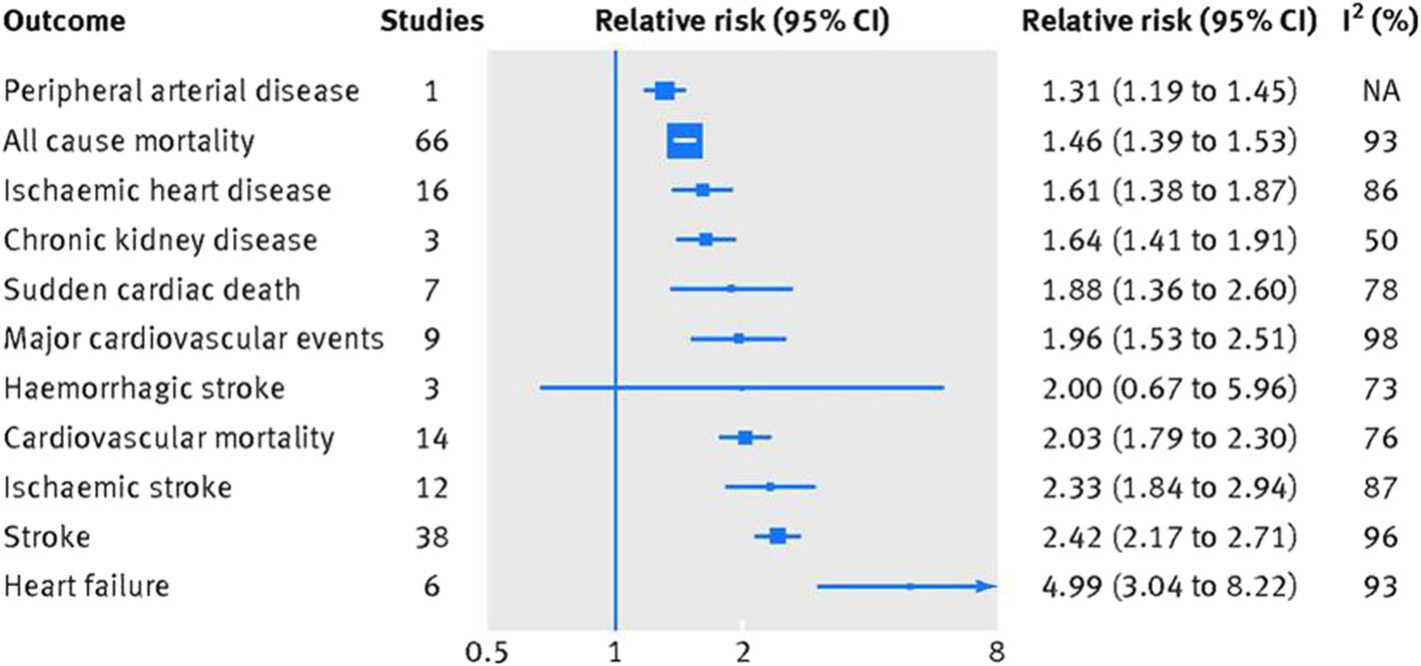
Association between atrial fibrillation and the all-cause mortality and cardiovascular and renal disease, with a summary of the relative risks of each outcome examined (cited from reference [41])

Both AF and HF are well known to have a reciprocal causal relationship, promoting the activities of each other, and together are associated with a significant increase in mortality and morbidity. A recent meta-analysis using 104 eligible cohort studies involving approximately one million participants [41] demonstrated that AF is associated with an increased risk of mortality (both all-cause mortality and cardiovascular mortality, including SCD), major cardiovascular events, ischemic strokes, IHD, HF, chronic kidney disease, and peripheral arterial disease (Fig. 6). Among those endpoints, the highest absolute risk increase was observed for HF with a relative risk of up to 4.99 (CI 3.04–8.22). In terms of the stage of AF, new-onset AF is associated with HF progression with a greater degree than chronic AF [42].

### AF associated with an acute MI

**Fig. 7:**
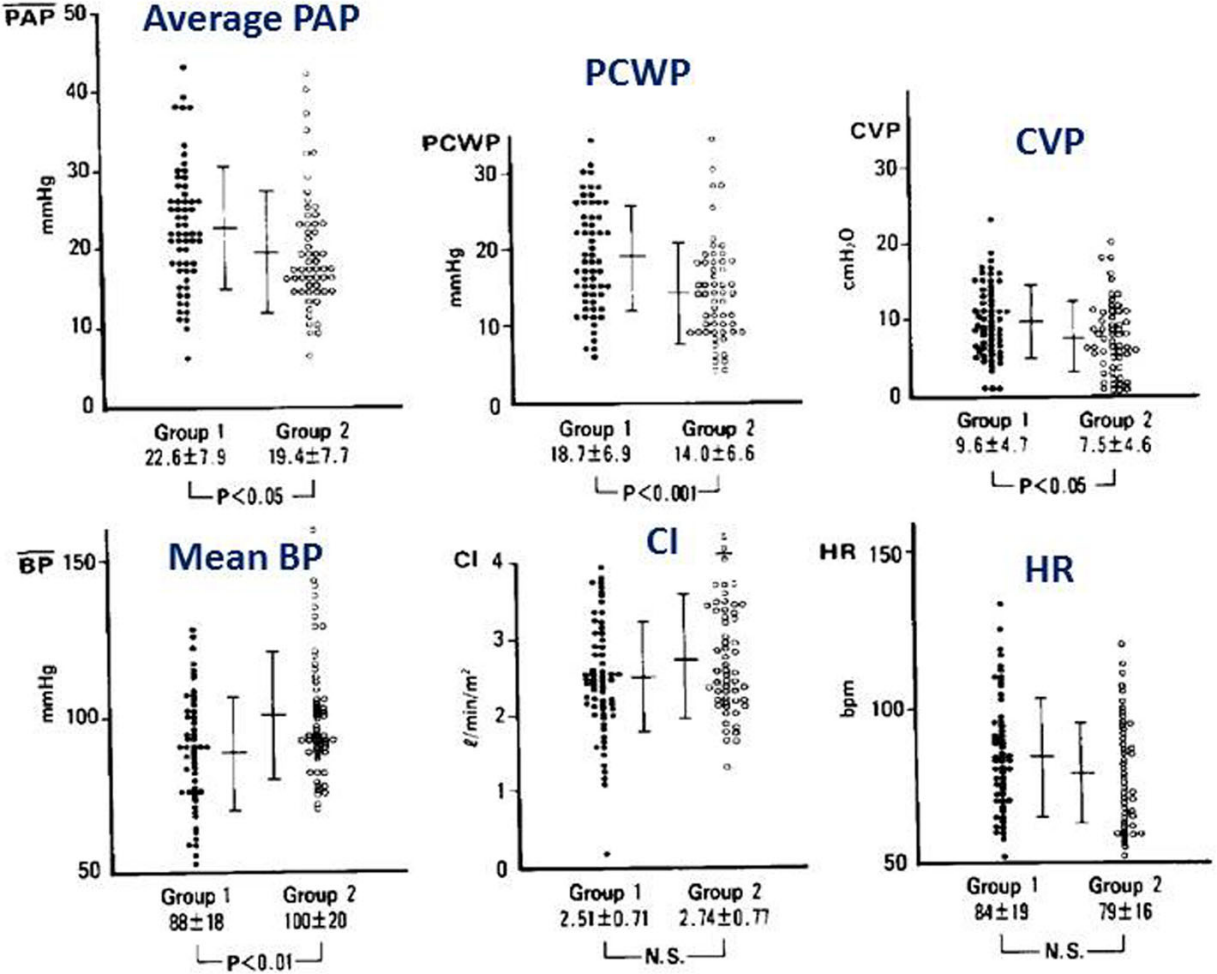
Comparative presentation of the hemodynamic variables between the patients with PAF (group 1) and those without PAF (group 2). The variables were measured during sinus rhythm, within 24 h before the onset of the PAF in group 1, and at the time of admission prior to various therapeutic interventions in group 2 (cited from reference [46]). PAP pulmonary artery pressure, PCWP pulmonary capillary wedge pressure, CVP central venous pressure, CI cardiac index, HR heart rate

Paroxysmal AF (PAF) occurs in 5 to 20% during the acute phase of an MI [43–46]. The development of PAF is facilitated by a variety of factors including (1) anatomic factors: ischemia of atrial structures (sinus node, AV node, and the atrial musculature) and pericardial effusions (pericarditis), (2) autonomic factors: an enhanced vagal tone accompanying an infero-posterior infarction and a sympathomimetic reaction in patients with a severe infarction, (3) hemodynamic factors: “pump failure” with left atrial hypertension, and (4) iatrogenic factors: digitalis, antiarrhythmic drugs, and sympathomimetic agents [47]. Among those factors, the most important factor underlying PAF is pump failure associated with a broad and severe MI [43–46]. Figure 7 shows comparative presentations of the hemodynamic variables between the patients with PAF (group 1) and those without PAF (group 2). Those variables were measured during sinus rhythm in both groups, within 24 h before the onset of PAF in group 1 and upon admission prior to various therapeutic interventions in group 2. Group 1 had a significantly higher pulmonary capillary wedge pressure (PCWP), higher central venous pressure (CVP), and lower blood pressure than group 2 [46]. It has been also shown that post-MI patients with a new onset of AF have a higher in-hospital mortality than those without AF. Moreover, AF itself is one of the independent predictors of a poor prognosis [43–45]. Therefore, it is recommended that treatment should be directed toward the mechanism producing the arrhythmia (mostly pump failure) in patients with PAF, and the treatment should be simultaneously directed at terminating or controlling the arrhythmia.

### Inflammation: sepsis

Recently it has been shown that AF commonly appears in critically ill patients with certain conditions, such as severe sepsis [47]. About 33% of critically ill patients with sepsis have AF, and 10% have new onset of AF [48]. Several pathogenic factors triggered by inflammation, such as hemodynamic compromise, cardiac injury, ischemia, and catecholamine surges, may promote the arrhythmia substrate. New onset of AF during a critical illness seems to be a marker of a poor prognosis, although there is no high-level evidence of this hypothesis [48]. Because there is little data in terms of how to manage AF in this category of patients, the management of AF in severely septic patients should be determined by a case-by-case fashion [49]. It is recommended that potentially reversible AF drivers, such as an electrolyte disturbance, acidemia, beta-agonist medications, and hypoxia, be promptly found, and those predisposing factors should be resolved.

### Management of AF in the critical care patients

Table 6 shows Japan’s guideline for the treatment of AF [37] associated with an acute MI, which is quoted from the ACC/AHA/ESC practical guidelines [50].

**Table 6:** Recommendations in the management of atrial fibrillation in acute myocardial infarction

CLASS I
1. Direct-current cardioversion is recommended for patients with severe hemodynamic compromise or intractable ischemia, or when adequate rate control cannot be achieved with pharmacological agents in patients with acute Ml and AF or AFL. *(Level of Evidence: C)*
2. Intravenous administration of amiodarone is recommended to slow a rapid ventricular response to AF and improve LV function in patients with acute Ml. (*Level of Evidence: C*) (*Out of insurance coverage*)
3. Intravenous beta blockers and nondihydropyridine calcium antagonists are recommended to slow a rapid ventricular response to AF in patients with acute Ml who do not display clinical LV dysfunction, bronchospasm, or AV block. *(Level of Evidence: C)*
4. For patients with AF and acute Ml, administration of unfractionated heparin by either continuous intravenous infusion or intermittent subcutaneous injection is recommended in a dose sufficient to prolong the activated partial thromboplastin time to 1.5 to 2.0 times the control value, unless contraindications to anticoagulation exist. *(Level of Evidence: C)*
CLASS lla
Intravenous administration of digitalis is reasonable to slow a rapid ventricular response and improve LV function in patients with acute Ml and AF associated with severe LV dysfunction and HF. *(Level of Evidence: C)*
CLASS III
The administration of class IC antiarrhythmic drugs is not recommended in patients with AF in the setting of acute Ml. *(Level of Evidence: C)*

When patients have a severe hemodynamic compromise or intractable ischemia, or when adequate rate control cannot be achieved with drug therapy, a direct-current (DC) cardioversion is recommended. Initially, a 200-J monophasic current or 120–200-J biphasic current is applied. If that is not successful, then energy current is increased by 50–100 J in a stepwise fashion. However, we sometimes experience new-onset AF with a very rapid ventricular response and hemodynamic deterioration that is refractory to DC cardioversion with the highest energy due to either failure of cardioversion to convert to sinus rhythm or an immediate re-initiation of AF. In this situation, the intravenous administration of class III drugs such as nifekalant can improve the patient outcome by decreasing the heart rate without decreasing the blood pressure during AF [51]. Moreover, nifekalant is shown to terminate AF and raise the success rate of DC cardioversion in some patients probably by reducing the defibrillation threshold.

For the rate control of AF to stabilize the hemodynamics, beta blockers and non-dihydropyridine calcium channel antagonists are used in patients who did not have either LV dysfunction or AV block. Due to the negative inotropic effects, these drugs are often intolerable in patients with HF. Intravenous amiodarone is a reasonable drug to improve this condition in that amiodarone may be expected to provide adequate rate control effects without any hemodynamic disturbance; however, the use of this drug for this aim is currently not under insurance coverage.

After the patient’s condition stabilizes, we should carefully consider the need for rhythm control therapy and anticoagulation therapy. In a clinical randomized trial in patients with AF and congestive HF (AF-CHF), the rhythm control strategy using antiarrhythmic agents and electrical cardioversion did not improve the all-cause mortality or prevent worsening HF as compared to a rate control strategy [52]. The reason was considered to be that the side effects and proarrhythmic risk from antiarrhythmic drugs may offset any salutary effects from restoring and maintaining sinus rhythm [53]. Catheter ablation of AF has been demonstrated to decrease the mortality and hospitalization and to improve the quality of life as compared to pharmacological therapy mainly with amiodarone in patients with a severely reduced LV function [54].

### Different diagnoses of tachycardia-induced cardiomyopathy (TICM) and tachycardia-mediated cardiomyopathy (TMCM) in AF patients

**Fig. 8:**
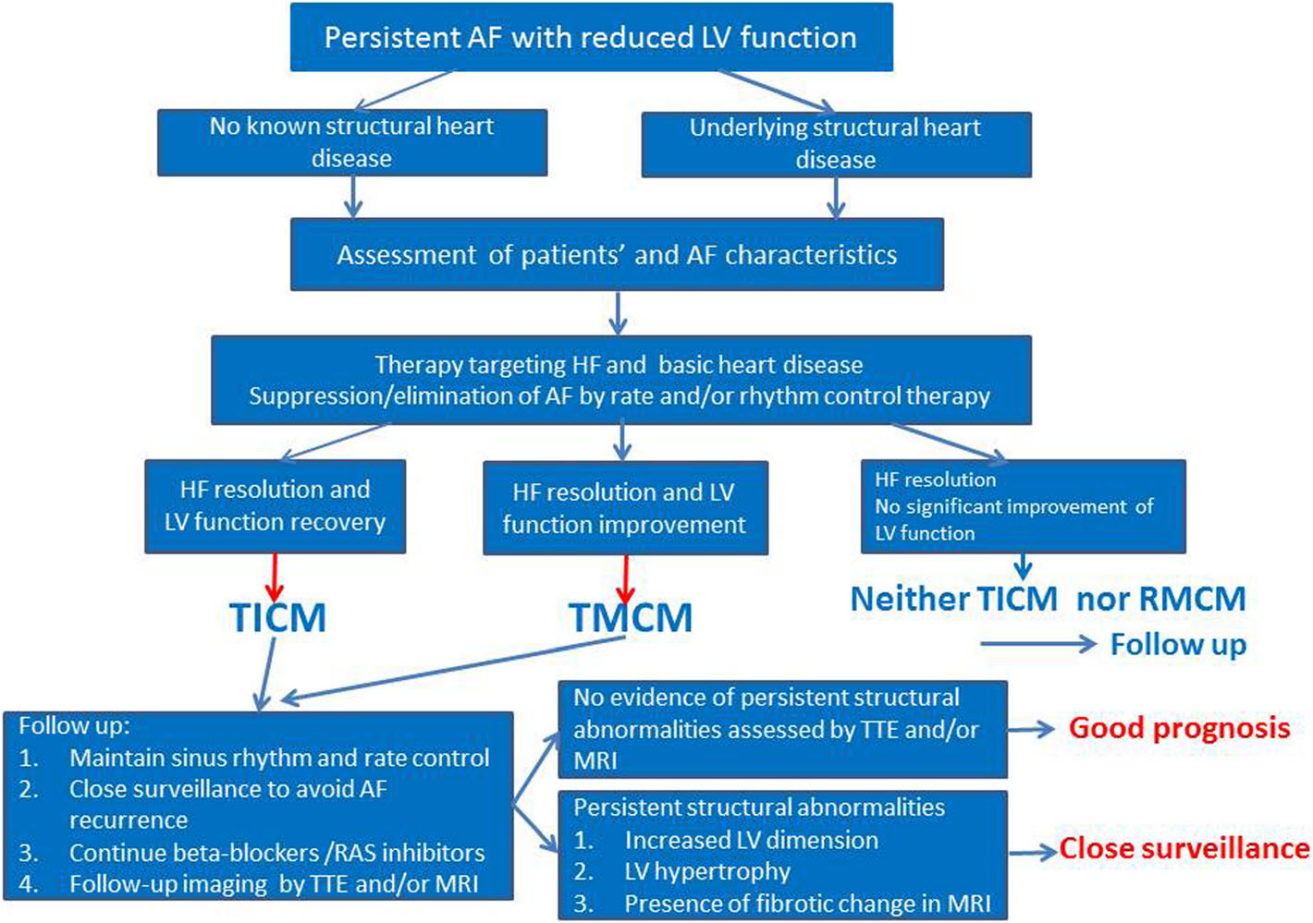
A diagnostic and therapeutic flowchart of the follow-up in the patients with tachycardia induced cardiomyopathy. For further details, see the text. TICM: tachycardia induced cardiomyopathy. TMCM: tachycardia-mediated cardiomyopathy (this figure was modified from Fig. 1 of reference [57])

In clinical practice, we often see the patients with both persistent AF and significantly reduced LV function that might be associated with a rapid ventricular response, the so-called tachycardia-induced cardiomyopathy (TICM). In this condition, a disturbance in the LV contraction is commonly normalized by an adequate rate control therapy [55, 56]. In TICM patients, early recognition of the relationship of the culprit arrhythmia to a reduced LV function is paramount in selecting a suitable treatment, which is likely to improve the patient’s condition. Figure 8 shows a diagnostic and therapeutic flowchart with the follow-up in the patients with TICM [57]. TICM can be classified into two categories, one in which the arrhythmia is the sole reason for the ventricular dysfunction (TICM) and another one in which the arrhythmia exacerbates the ventricular dysfunction and/or worsens the HF in patients with concomitant structural heart disease (TMCM). In either situation, the treatment modalities should be selected on a case-by-case basis targeting both the HF and AF itself. Then, if the HF resolves and LV function totally recovers, the patient can be diagnosed with TICM. When the HF resolves and the LV function somehow improves, it is confirmed to be TMCM. Finally, if there is no significant improvement in the LV function, it is neither TICM nor TMCM (see Fig. 8) [57]. Close surveillance is recommended in these patients, because the recurrence of AF can result in a rapid decline in the cardiac performance, even after normalization of the LV function by the initial treatment, and because there are several patient reports of SCD even in HF patients related to AF [55]. Since it has been shown that it takes 1–6 months for a complete recovery of the cardiac function [55, 56], the cardiac function should be re-evaluated using transthoracic echocardiography after a corresponding interval.

## Risk stratification of sudden cardiac death and prevention therapy

After the critical management of patients with various cardiac diseases, the risk of sudden cardiac death (SCD) should be stratified for each patient to assess the individual need for preventive therapies. These are cardiac implantable electrical devices (CIEDs) incorporated with the function of an ICD, including cardiac resynchronization therapy defibrillators (CRT-Ds) and wearable ICDs.

### Risk stratification of SCD in coronary artery disease

During the acute phase of an MI, the clinical significance of documented VTAs in terms of the predictive value of a future appearance of lethal VTAs varies as the time lapses [58, 59]. Non-sustained VT (NSVT) or VF, which emerge within 48 h from the onset of the MI, do not necessarily predict the future occurrence of lethal arrhythmias. Although NSVT, which occurs after 24  h from the onset of an MI [60], has been shown to be a significant predictor of severe VTAs, the early application of an ICD after an MI does not improve the patient’s prognosis [61, 62]. The benefit of an ICD (decrease in arrhythmic death) is offset by the increase in HF death, which is presumably caused by either appropriate or inappropriate shock deliveries [61]. Therefore, high-risk MI patients with SCD, such as those with a low left ventricular ejection fraction (LVEF) of ≤ 35%, should be monitored for at least 1 month after the onset of the MI according to the guidelines of the Japan Circulation Society [63]. If the patients have a transient high risk of lethal arrhythmias, a wearable ICD is recently indicated [64]. It can be expected to play a role as a bridge therapy to an ICD implantation and help the LV function recovery in patients at high risk for lethal VTAs, particularly during the acute phase of an MI and after a percutaneous coronary intervention (PCI). In patients with a remote MI, an ICD should be introduced if VF is clinically documented. Polymorphic VT, monomorphic (mono-) VT with hemodynamic compromise, drug refractory mono-VT, and mono-VT, which cannot be cured by catheter ablation, are also class I as indications for an ICD [63]. With regard to primary prevention, the criteria for a class I indication of an ICD includes an NYHA class II or III with an LVEF ≤ 35% and NSVT under optimal medical therapies. In addition, an NYHA class I with an LVEF ≤ 35%, NSVT, and inducible sustained VTAs during the electrophysiological studies is another class I indication. On the other hand, the SCD-HeFT criteria in which only an NYHA class (II or III) and LVEF ≤ 35% are essential conditions are a class IIa indication. This is an important difference from the AHA/ACC/ESC guidelines [65] in which the SCD-HeFT criteria are ranked as a class I indication [45]. Recently, a report from the CHART-2 study [66] demonstrated that the prevalence of fatal arrhythmic events was 16.1% in patients categorized into a class I indication with the Japanese Circulation Society (JCS) guidelines, 8.9% in those with a class IIa indication, and 1.9% for those with no indication. Thus, the current JCS guidelines for prophylactic ICD usage have been validated at least for Japanese patients with CHF. Antiarrhythmic medical therapy (amiodarone, sotalol, and beta blockers) and catheter ablation are currently considered to be supplemental therapies for the reduction of appropriate and inappropriate shock deliveries by the ICD [67, 68]. Both shock therapies have been shown to aggravate the patient’s prognosis [69, 70].

### Risk stratification of SCD in non-ischemic cardiomyopathy

For patients with DCM, the risk stratification and indication of an ICD are analogous to that for a remote MI; however, the clinical significance of an inducible VTA in the risk stratification and the effect of the ICD in terms of mortality reduction are less in DCM as compared to IHD [63]. In the risk assessment for primary prevention in HCM, the thickness of the interventricular septum (≥ 30 mm), family history of SCD, an abnormality in the blood pressure response during exercise, and the presence of NSVT are important markers of a poor prognosis [71]. These are the essential conditions for a class IIa indication for an ICD in the JCS guidelines [63].

### Risk stratification of SCD in the inherited channelopathies

This category includes long QT syndrome, idiopathic VF (Brugada syndrome, early repolarization syndrome, and short-coupled variant of torsade de points), catecholaminergic polymorphic ventricular tachycardia (CPVT), and short QT syndrome. Japan’s guideline for ICD indication is currently available for long QT syndrome and Brugada syndrome. In brief, for the long QT syndrome, a history of either VF or cardiac arrest is class I indication of ICD, while a history of syncope and/or torsade de points which is refractory to beta blockers is class IIa indication. For Brugada syndrome, a history of aborted cardiac arrest and a documentation of VF or polymorphic VT are class I indications for ICD, while the patients with spontaneous coved type ST elevation in precordial leads who meet at least two criteria out of the following three criteria (history of syncope, family history of sudden cardiac death, and inducibility of VF by EP testing) (for details, please see the JCS guideline) [63, 72]. At the present time, the diagnostic role of the localization of responsible gene mutation is important; however, its role for the risk stratification remains unclarified.

## Conclusion

In the practice of cardiovascular critical care, we often meet a variety of arrhythmias with a variety of clinical backgrounds. We should pay attention not only to the characteristics and mechanisms of the existing arrhythmias, but also to the upstream pathophysiology underlying the occurrence of those arrhythmias. We also have a lot of therapeutic options for the treatment of arrhythmias that often suppress them and improve the patient status. However, conversely, those therapies sometimes bring harmful results. Therefore, we should judge the necessity of the suppressive treatment of arrhythmias and select the most appropriate modality of treatment on a case-by-case basis.

## Abbreviations

AF: Atrial fibrillation
APC: Atrial premature contraction
CCU: Coronary care unit
CIEDs: Cardiac implantable electrical devices
CS: Cardiac sarcoidosis
ES: Electrical storm
HF: Heart failure
HFpEF: Heart failure with preserved ejection fraction
HFrEF: Heart failure with reduced ejection fraction
ICD: Implantable cardioverter defibrillator
ICU: Intensive care unit
IHD: Ischemic heart disease
IVT: Idiopathic ventricular tachycardia
LQTS: Long QT syndrome
MI: Myocardial infarction
NSVT: Non-sustained ventricular tachycardia
PM: Pacemaker
SCD: Sudden cardiac death
SND: Sinus node dysfunction
SVT: Supraventricular tachycardia
Tdp: Torsade de pointes
TICM: Tachycardia-induced cardiomyopathy
TMCM: Tachycardia-mediated cardiomyopathy
VF: Ventricular fibrillation
VPC: Ventricular premature contraction
VT: Ventricular tachycardia
VTA: Ventricular tachyarrhythmia
