# Correction to: How to manage various arrhythmias and sudden cardiac death in the cardiovascular intensive care

**DOI:** 10.1186/s40560-018-0300-1

**Published:** 2018-05-24

**Authors:** Yoshinori Kobayashi

**Affiliations:** Division of Cardiology, Tokai University Hachioji Hospital, 1838, Ishikawa-machi Hachioji-shi, Tokyo, 192-0032 Japan

## Correction

The author noted that major part of Acknowledgements section was inadvertently missing in the original article [1]. The new Acknowledgements can be found below.

“I am grateful to Morimasa Takayama M.D., the President of Tokyo CCU Network, the Vice-director of Sakakibara Heart Institute, for his expert insight and advice in the ischemia-associated mechanisms of ventricular arrhythmias in acute myocardial infarction, and to Ken Nagao M.D., the Chairman of the scientific committee, Tokyo CCU Network, Division of Cardiology, Nihon University Hospital, for his great suggestions in the scientific interpretation of the large data from Tokyo CCU Network. I also express a special gratitude to all the member hospitals of Tokyo CCU Network for their great contributions to the registry study of acute myocardial infarction in the Tokyo metropolitan area. Finally, I thank Mr. John Martin for his linguistic assistance”.
